# The Importance of Early Detection and Minimally Invasive Treatment of Pseudoaneurysms Due to Chronic Pancreatitis: Case Report

**DOI:** 10.3390/medicina60050714

**Published:** 2024-04-26

**Authors:** Dejan Velickovic, Katarina Stosic, Aleksandra Djuric Stefanovic, Jelena Djokic Kovac, Danijela Sekulic, Stefan Milosevic, Marko Miletic, Dusan Jovica Saponjski, Borivoje Lukic, Boris Tadic, Milica Mitrovic Jovanovic, Vladimir Cvetic

**Affiliations:** 1 Department of Stomach and Esophageal Surgery, Clinic for Digestive Surgery, University Clinical Centre of Serbia, Koste Todorovica Street No. 6, 11000 Belgrade, Serbia; 2 Department for Surgery with Anesthesiology, Faculty of Medicine, University of Belgrade, Dr Subotica No. 8, 11000 Belgrade, Serbia; 3 Center for Radiology and Magnetic Resonance Imaging, University Clinical Centre of Serbia, Pasterova No. 2, 11000 Belgrade, Serbia; 4 Department for Radiology, Faculty of Medicine, University of Belgrade, Dr Subotica No. 8, 11000 Belgrade, Serbia; 5 Department for HPB Surgery, Clinic for Digestive Surgery, University Clinical Centre of Serbia, Koste Todorovica Street, No. 6, 11000 Belgrade, Serbia

**Keywords:** chronic pancreatitis, pseudoaneurysm, pancreatic pseudocyst, walled-off necrosis

## Abstract

The occurrence of the pseudoaneurysm of visceral arteries in the field of chronic pancreatitis is a very rare complication that represents a life-threatening condition. The higher frequency of this complication is in the necrotic form of pancreatic inflammation, especially in patients with formed peripancreatic necrotic collections. The degradation of the arterial wall leads to bleeding and transforms these necrotic collections into a pseudoaneurysm. Urgent endovascular angioembolization is the first choice in the therapeutic approach as a valid minimally invasive solution with very satisfactory immediate and long-term outcomes. This successfully avoids open surgery, which is associated with a high mortality rate in these patients, especially in acute-on-chronic pancreatitis.

## 1. Introduction

Chronic pancreatitis is an inflammatory disease of the pancreas characterized by repeated bouts of acute pancreatitis and subsequent fibrosis, often resulting in various complications, including vascular ones [1]. Visceral artery pseudoaneurysms represent a well-known complication of pancreatitis, which occurs due to the proteolytic degradation of the arterial wall giving rise to a pseudoaneurysm formation [2].

The incidence of pseudoaneurysms in patients with pancreatitis is around 10%, most commonly affecting the splenic artery in 50% of cases, followed by the gastroduodenal (10%) and pancreaticoduodenal (10%) arteries [3]. Left gastric artery pseudoaneurysm is rare, with less than 50 cases reported in the literature [4].

Pseudoaneurysms might be asymptomatic in 7.5% cases, but may also have symptoms like mild epigastric discomfort or pain and a palpable pulsating mass. On the other end of the spectrum, a serious clinical course can be seen if they penetrate into the main pancreatic duct, followed by gastrointestinal bleeding, or after spontaneous rupture. The signs of a ruptured pseudoaneurysm include anemia, melena, hematemesis due to bleeding through the Wirsung duct and papilla Vateri into the duodenum or directly into the jejunum through the bedsore of its wall due to the pseudoaneurysm, and massive intraperitoneal hemorrhage with severe abdominal pain and hypovolemia. The mortality of a ruptured visceral artery aneurysm is high, up to 40%, thus requiring a prompt diagnosis and management [2].

During the evolution of “walled-off” pancreatic necrosis (WON), the adherence of the surrounding vessels to the wall of the WON might occur, causing thrombosis in the veins and damage of the thick arterial wall with proteolytic enzymes forming a pseudoaneurysm. Contrary to true aneurysms which contain all layers of the arterial wall, a pseudoaneurysm wall is made of fibrotic tissue with a superimposed hematoma, making it prone to enlargement or rupture. The incidence of massive hemorrhage leading to hemodynamic instability as a consequence of a ruptured pseudoaneurysm formed on the grounds of chronic pancreatitis is around 1.4% to 8.4% according to the literature, with an exceedingly high mortality rate of up to 90% [4,5].

We report a rare case of a left gastric artery pseudoaneurysm in a patient with chronic pancreatitis which was diagnosed at the very beginning of its formation, confirmed at follow-up imaging, with a successful treatment by minimally invasive endovascular embolization. Written informed consent was obtained from the patient.

## 2. Case Report

The reporting of this study conforms to the CARE guidelines [6]. A 54-year-old male patient was admitted to our hospital due to epigastric pain that lasted for a month. His previous medical history included chronic pancreatitis and alcohol abuse.

At the time of admission, a clinical examination revealed diffuse tenderness, especially in the epigastric area. The patient was afebrile with normal vitals.

Laboratory examinations, including a complete blood count, showed a normal number of white blood cells and an elevated C-reactive protein level 64.1 mg/L. The biochemistry test results were suggestive of acute pancreatitis, with high amylase (609 U/L) and lipase (504 U/L) levels.

**Figure 1:**
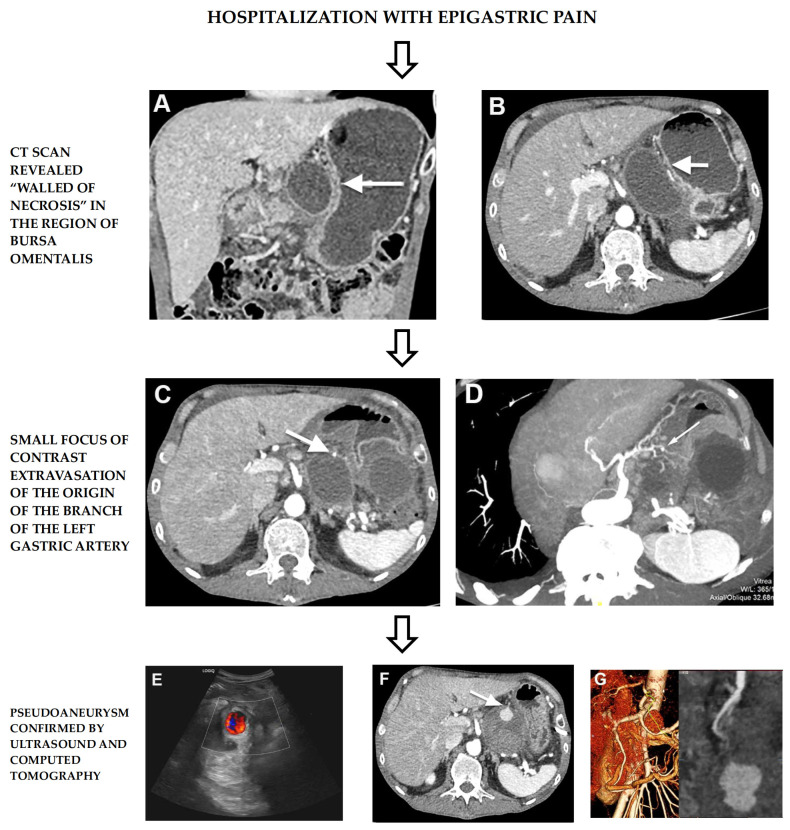
Well-defined cystic mass in the region of bursa omentalis—WON (**A**,**B**). Small focus of contrast extravasation suspected on pseudoaneurysm formation (**C**,**D**). Yin-yang sign on abdominal ultrasound with CD corresponding to turbulent blood flow in formed pseudoaneurysm (**E**). CT presentation of pseudoaneurysm originating from left gastric artery within WON (**F**,**G**).

An initial abdominal contrast-enhanced computed tomography scan (CT) revealed a round encapsulated thin-walled cystic mass with a maximum diameter of 7.5 cm in the region of the pancreatic body and tail extending into the bursa omentalis (Figure 1A,B). In addition, multiple calcifications and smaller cysts were found in the head and body of the pancreas, as signs of chronic pancreatitis. Those findings were indicative of acute necrotic pancreatic inflammation on the grounds of chronic pancreatitis.

The patient was treated with conservative therapy that included adequate fluid resuscitation, antibiotic prophylaxis, and parenteral analgesics.

After ten days, due to sudden severe pain, the patient underwent a new abdominal contrast-enhanced CT examination that showed a persistent and slightly enlarged well-defined round, necrotic collection corresponding to WON in the region of the bursa omentalis. In the anterior wall of the collection, a small focus of contrast extravasation was seen in the arterial phase which raised the suspicion of pseudoaneurysm formation (Figure 1C,D); therefore, a strict follow-up was scheduled.

A control abdominal ultrasound examination (US) was performed after 7 days, which showed a collection in the region of the pancreatic body with an internal Color Doppler (CD) signal. The CD signal was bidirectional due to turbulent blood flow, indicating the presence of a formed pseudoaneurysm (yin-yang sign) (Figure 1E). A CT examination confirmed the existence of a pseudoaneurysm at the site of the previously seen small focus of contrast extravasation (Figure 1F,G). Then, 3D Volume Rendering CT showed a central enhancing component surrounded with necrotic tissue, that indicated a pseudoaneurysm originating from the distal branch of the left gastric artery (Figure 1G).

**Figure 2:**
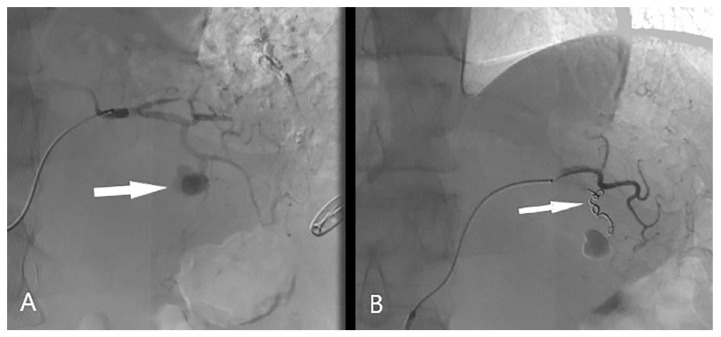
Supra-selective catheterization of left gastric artery showing a pseudoaneurysm (white arrow) of one of the distal branches (**A**) Post-embolization digital subtraction angiography showing that the pseudoaneurysm is excluded from the circulation. (**B**) The image shows 3 mm × 5 cm coil (white arrow) in the distal part of the left gastric artery branch.

Considering the patient’s condition, and the location and size of the pseudoaneurysm, digital subtraction angiography (DSA) with endovascular coil embolisation was performed. A right retrograde transfemoral approach was used for the selective catheterization of the celiac trunk, which was performed with a 5-French Cobra-C2 diagnostic catheter (Terumo Corporation, Tokyo, Japan). Afterward, the supraselective catheterization of the branch of the left gastric artery with a 2,4-French microcatheter (Progreat Microcatheter System, Terumo, Japan) was carried out. The angiographically visualized pseudoaneurysm of one of the branches of the left gastric artery (Figure 2A) was embolized. The Azure 18 Detachable 3 mm/5 cm Helical Hydrocoil (Microvention Inc. Tustin, CA, USA) was placed in the distal segment of the branch (Figure 2B).

The day after the procedure, the patient had transient shivers and chills, which disappeared with appropriate therapy. A follow-up US showed the absence of a CD signal in the liquid collection. The patient was discharged in a good general condition three days after the procedure. A control US after one month showed a significant reduction in the collection in the omental bursa.

## 3. Discussion

Chronic pancreatitis (CP) manifests as a progressive inflammation of the pancreas with subsequent fibrosis, irreversible structural changes, and a deficiency in its function. It is most frequently attributable to alcohol abuse but can also occur due to hereditary and autoimmune pancreatitis, systemic diseases, and idiopathic causes [1].

In 20–40% of patients, an acute exacerbation of chronic pancreatitis leads to the renewed inflammation of the pancreas and peripancreatic tissue, which can be complicated by the formation of acute liquid or necrotic collections, i.e., the formation of pseudocyst or “walled-off” pancreatic necrosis [7,8].

An acute necrotic collection represents an aggressive inflammatory process and involves the pancreatic parenchyma, sometimes with an extension to the peripancreatic fatty tissue. In fact, it is a collection of dense necrotic fluid that may contain the foci of a hemorrhage—hemorrhagic pancreatic necrosis. After four weeks, it tends to organize, with a thicker wall in the form of WON [9,10].

Patients with walled-off necrosis should be treated individually and monitored closely by a multidisciplinary team, as various complications may occur throughout the disease evolution [10].

Most common complications of chronic pancreatitis include jaundice caused by biliary obstruction, duodenal obstruction due to an inflammatory mass or fibrosis, splenic vein thrombosis, and pseudoaneurysm formation [1].

Visceral artery pseudoaneurysms represent a rare complication which can occur in both chronic pancreatitis with an incidence of 4–10% and acute pancreatitis with a lower incidence of 1–6%. However, they are potentially life-threatening conditions as the mortality rate is up to 40% and, if left untreated, can reach as high as 90% [11,12]. They are considered to be caused either by the digestion of the pancreas and its surrounding tissue by lipolytic and proteolytic pancreatic enzymes, causing injury to the arterial wall, or by the erosion and conversion of a pseudocyst/WON [11,12]. Further, a formed pseudoaneurysm within the pancreatic necrosis may perforate within the digestive tract, with melena or hematemesis as the first sign of this complication [10,12].

More frequently, they are found in patients with the etiology of alcohol abuse (80%) [12].

The splenic artery is the most common site affected by this process (30–50% of cases), followed by the gastroduodenal (10–15%) and pancreaticoduodenal artery (10%); the isolated involvement of the left gastric artery, as in our case, is a very rare condition [13,14].

Our exploration of the Medline database (PubMed) has unveiled fewer than 50 instances of left gastric artery pseudoaneurysms. The scarcity of such pseudoaneurysms is evident in the research conducted by Vujasinovic et al., where, out of 394 chronic pancreatitis patients, only 33 exhibited vascular complications, with merely one case involving a pseudoaneurysm of the left gastric artery [15]. Similarly, Pitton et al.’s study, encompassing 253 patients, reported a mere three cases of right and left gastric artery pseudoaneurysms [16]. Even in pediatric investigations of pancreatitis by Chinenye et al., comprising 410 patients, only a solitary pseudoaneurysm of the left gastric artery was identified [17].

As 2.5% of bleeding pseudoaneurysms can be clinically inconspicuous, the rigorous follow-up of patients at high risk is mandatory, with imaging modalities being of the utmost importance for establishing a prompt diagnosis [18].

The pseudoaneurysms are mainly diagnosed on a computed tomography angiography (CTA) as it has a high sensitivity and specificity and can demonstrate in detail the size of the aneurysm and its neck, and its relation to the surrounding structures, as well as partial thrombosis and lumen patency [19]. A post-processing analysis with maximum intensity projections and 3D reconstructions with a vessel analysis on CTA allows detailed pretreatment planning. The signs of pseudoaneurysm formation on CTA are commonly as follows: an inhomogeneous hyperdense lesion on the precontrast evaluation, which enhances avidly in the arterial phase, and then, in the venous phase, slightly decreases in density; however, if it is not ruptured, the size remains the same. Sometimes, an adjacent hematoma can be seen due to leakage or rupture [13].

At times, a Dopler ultrasound can also aid in the detection of the pseudoaneurysmal sac, which is classically represented as a hypoechoic cystic structure in the vicinity of an artery, typically demonstrating the “yin-yang sign” (bidirectional, back-and-forth flow of blood from the neck into the lumen of the pseudoaneurysm following cardiac cycle) [13].

Rarely, as shown in this case, the beginning of a pseudoaneurysm formation can be seen as a small, contrast-filled outpouching arising next to the branch of a left gastric artery. Risk factors for pseudoaneurysm formation include chronic pancreatitis where alcoholism is the underlying etiology, as well as presence of fluid collections, i.e., pseudocyst or WON [10]. Usually, in such patients, melena and hematemesis appear in the clinical course of the disease due to rupture occurrence and upper gastrointestinal bleeding, as Gaur et al. described in their article [20]. However, this was not the case with our patient.

To the best of our knowledge, no previous papers have shown the course of the development of a pseudoaneurysm from its very beginning as a small barely notable outpouching in the proximity of a branch of a left gastric artery to the fully formed pseudoaneurysm within a “walled-off” pancreatic collection. In addition, the left gastric artery is a very rare place of origin for a pseudoaneurysm, and several cases have been published in the literature so far [20].

The timely detection of the pseudoaneurysm in these patients is of great importance because, according to the literature, the precise criteria for a rupture are not clearly defined, but it is certain that re-acute inflammation would contribute to a higher risk of rupture and hemorrhage.

Visceral artery aneurysms require prompt management as they have proven to be inherently unstable, with a high risk of rupture and hemorrhage which is unrelated to the size of the pseudoaneurysm, as well as a high mortality rate of up to 40%, depending on the severity and duration of the underlying pancreatitis [14].

The decision to monitor or intervene with visceral artery aneurysms depends on the artery affected, its size, and the underlying disease [19].

The management of ruptured visceral artery aneurysms is aimed at controlling life-threatening hemorrhage with vigorous resuscitation and definitive intervention [19]. After the evaluation of cross-sectional imaging and according to all above-mentioned CT findings that indicate a pseudoaneurysm formation, an angiographic examination should be promptly performed. A selective angiogram should confirm the diagnosis and guide the possible embolization, as described by Varrassi et al. [13]. In our case, we were also guided by that protocol. Endovascular embolization is, nowadays, the treatment of choice for managing pseudoaneurysms in hemodynamically stable patients, since it is associated with a lower post-operative morbidity and mortality (4–19%) in comparison to surgical procedures, and a high rate of technical success (67–97%) [21]. It is usually performed with coils or glue during emergency procedures. If the patency of the vessel is required, it is also recommended to use covered stents. The detailed localization and characterization of the pseudoaneurysm on preprocedural imaging may guide the choice of the most suitable technique [19]. In our case, we opted for the embolization of both the inflow and outflow of the pseudoaneurysm with the sandwich technique using 0.014-inch detachable three-dimensional coils 3 mm × 5 cm for the embolization of the distal branch of left gastric artery, due to the fact that this blood vessel was terminal, without significant distal vascularization.

The post-intervention all-cause 30-day mortality has been reported as being between 6.2% and 6.7% [16,22].

The surgical resection of a peripancreatic visceral artery pseudoaneurysm has demonstrated a high mortality rate [11]. Consequently, it is performed only in hemodynamically unstable patients where angiography would be time-consuming. Fibrotic changes surrounding the pancreas hinder the surgical procedure and increase the mortality. Therefore, the surgical resection of the pancreas, especially in emergency settings, should be avoided, and, whenever possible, the ligation of the feeding artery could be performed [11]. Only when all other options are unsuitable should the pancreatic and peripancreatic tissue containing the pseudoaneurysm be resected.

## 4. Conclusions

Complications of necrotic pancreatitis, as we can conclude, can lead to a life-threatening condition. Peripancreatic necrosis represents a high risk factor for the occurrence of pseudoaneurysms of the surrounding arterial blood vessels. Bearing this in mind, the regular diagnostic follow-up of these patients is of great importance, as is the detection of all signs that indicate the possible occurrence or already present formation of a pseudoaneurysm, as well as the impending rupture. Minimally invasive endovascular angioembolization represents the gold standard in the therapeutic approach for these patients as a valid minimally invasive solution with very satisfactory immediate and long-term outcomes. If the patency of the visceral vessel is required, the use of a covered stent can be considered.

## Data Availability

No new data were created or analyzed in this study. Data sharing is not applicable to this article.
